# Supplementary material for: phox2ba: The Potential Genetic Link behind the Overlap in the Symptomatology between CHARGE and Central Congenital Hypoventilation Syndromes
Source: Genes (Basel). 2023 May 15;14(5):1086. doi: 10.3390/genes14051086 (PMC10218506; doi:10.3390/genes14051086)
Supplement: Supplementary file 1 [file genes-14-01086-s001.zip › genes-1996293-supplementary.pdf]

Supplementary Material

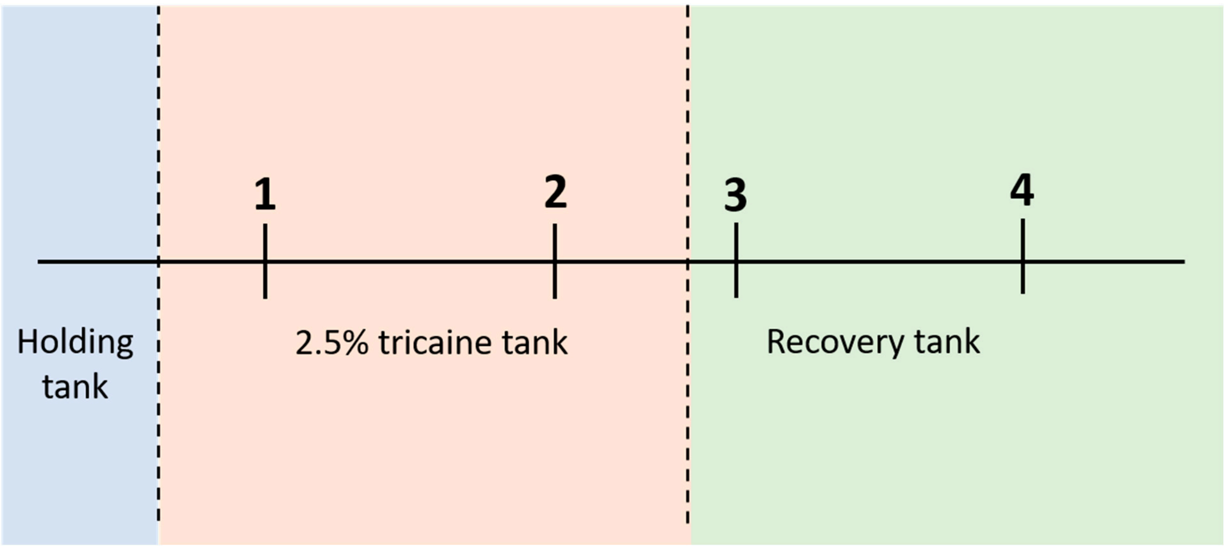

Supplementary Figure S1. Respiratory rate behavioural experimental timeline. Adult fish were transferred to a tank containing 2.5% tricaine and respiratory rates (opercular movements) were recorded at the time fish lost their response to touch (time point 1- post loss of touch response) and response to fin-pinch (time point 2- post loss of fin pinch response). Fish were then moved to a recovery tank, and respiratory rates were assessed both upon entering the recovery tank (time point 3- initial recovery tank) and just before fish recovered ability to swim (time point 4- 10 s prior to recovery). Note that videos were captured in real time and opercular beats were counted at a later point from the videos.

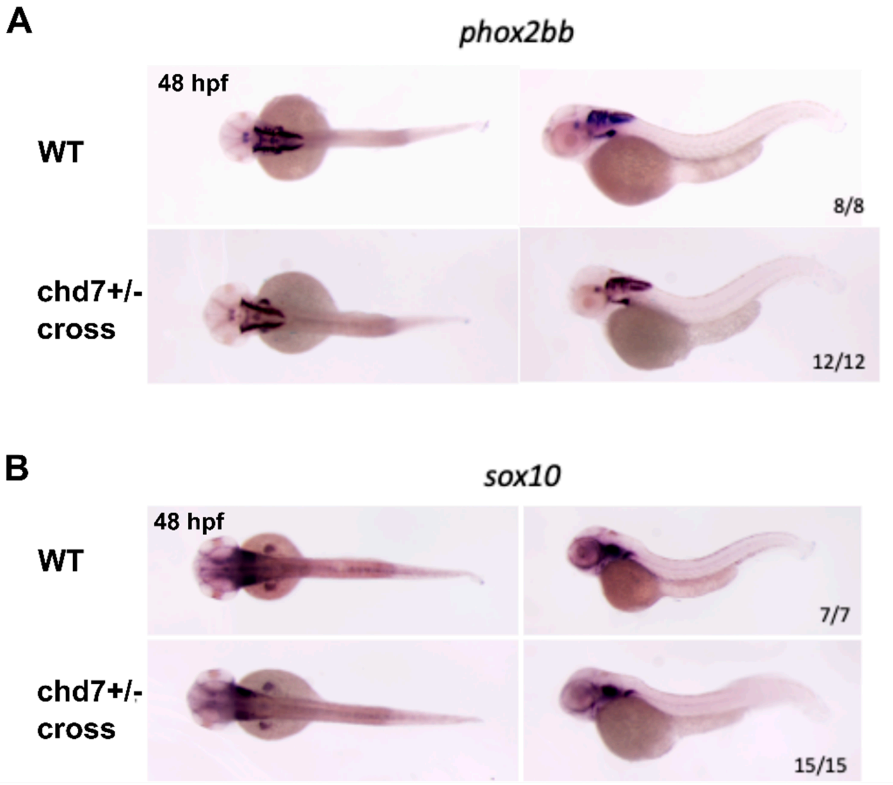

Supplementary Figure S2. ***phox2bb*** **and** ***sox10*** **expression levels appear similar in** ***chd7***$^{+/-}$ **incross and wild-type (WT) embryos.** (**A**) Representative images of *phox2bb* expression patterns in WT and *chd7*$^{+/-}$ cross embryos at 48 hours post fertilization (hpf). (**B**) Representative images of *sox10* expression patterns in WT and *chd7*$^{+/-}$ cross embryos at 48 hpf.

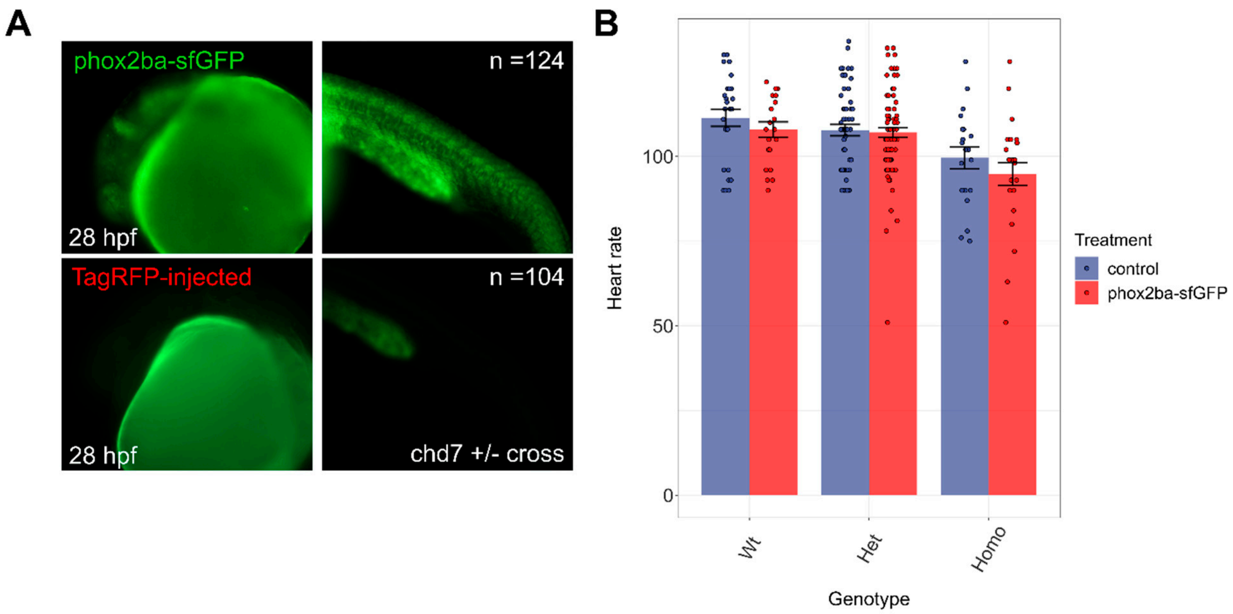

Supplementary Figure S3. Expression of phox2ba-sfGFP mRNA does not rescue reduced heart rate in chd7$^{-/-}$ **embryos at 3 dpf. (A)** Representative head and tail images of phox2ba-sfGFP mRNA injected chd7$^{+/-}$ cross embryos at 28 hpf (top row) and control chd7$^{+/-}$ cross embryos injected with TagRFP mRNA. Both were imaged in the EGFP channel. Total numbers of embryos analyzed from 3 independent experiments are indicated on the images Total numbers of respective embryos analyzed are indicated on the top of tail images. **(B)** Graph of heart rate quantifications at 3 dpf with respect to the genotypes and injections. Wt = chd7$^{+/+}$, Het = chd7$^{+/-}$, Homo = chd7$^{-/-}$.
